# Tocilizumab Trough Levels Variability in Kidney-Transplant Candidates Undergoing Desensitization

**DOI:** 10.3390/jcm11010091

**Published:** 2021-12-24

**Authors:** Aurélie Truffot, Thomas Jouve, Johan Noble, Béatrice Bardy, Paolo Malvezzi, Lionel Rostaing, Françoise Stanke-Labesque, Elodie Gautier-Veyret

**Affiliations:** 1 Laboratoire de Pharmacologie, Pharmacogénétique et Toxicologie, CHU Grenoble Alpes, 38043 Grenoble, France; atruffot@chu-grenoble.fr (A.T.); FStanke@chu-grenoble.fr (F.S.-L.); egautier@chu-grenoble.fr (E.G.-V.); 2 Service de Néphrologie, Hémodialyse, Aphérèses et Transplantation, CHU Grenoble Alpes, 38043 Grenoble, France; tjouve@chu-grenoble.fr (T.J.); jnoble@chu-grenoble.fr (J.N.); pmalvezzi@chu-grenoble.fr (P.M.); 3 Institute for Advanced Biosciences, UMR CNRS 5309/INSERM U1209, Université Grenoble-Alpes, 38400 Grenoble, France; 4 Etablissement Français du Sang (EFS Rhône-Alpes), Seteur HLA, 38043 Grenoble, France; Beatrice.Bardy@efs.sante.fr; 5 Université Grenoble Alpes, INSERM, CHU Grenoble Alpes, HP2, 38000 Grenoble, France

**Keywords:** monoclonal antibodies, desensitization, pharmacokinetic, kidney-transplant, tocilizumab

## Abstract

The presence of anti-HLA antibodies is an increasing challenge in kidney transplantation. Tocilizumab (TCZ), a monoclonal antibody targeting the interleukin-6 receptor (IL-6R), has been proposed to complement conventional desensitization therapy. We aimed to describe TCZ plasma trough concentrations and their variability and to investigate the link between TCZ concentration and the evolution of anti-HLA antibodies. Sensitized kidney-transplant candidates treated monthly with TCZ (8 mg/kg) for desensitization were retrospectively included. TCZ concentrations were determined by liquid chromatography-tandem mass spectrometry. Seventy-four TCZ concentrations from 10 patients were analyzed. The TCZ trough concentration ranged from <1.0 to 52.5 mg·L^−1^, with a median of 25.6 mg·L^−1^ [25th–75th percentiles: 13.2–35.3 mg·L^−1^). The inter- and intra-individual coefficients of variation were 55.0% and 33.0%, respectively. The TCZ trough concentration was not related to IL-6 (rho = −0.46, *p* = 0.792), soluble IL-6R (rho = −0.81, *p* = 0.65) concentrations or reduction of anti-HLA antibodies (mixed-effects model adjusting, effect of TCZ trough concentration: rho = −0.004, *p* = 0.26). The individual median TCZ concentration tended to be associated with the number of antibodies, with an initial MFI > 3000 that dropped to <3000 after TCZ treatment (rho = 0.397, *p* = 0.083). TCZ trough concentrations in kidney-transplant candidates treated for desensitization were highly variable. Further studies on larger cohorts are needed to study the possible link between TCZ concentrations and the reduction of anti-HLA antibodies.

## 1. Introduction

The presence of anti-HLA antibodies in patients awaiting a kidney-transplantation is a major limitation to successful transplantation, particularly in patients with panel-reactive alloantibodies (PRA) larger than 95%. Various strategies for desensitization, based notably on plasmapheresis and the administration of rituximab that sometimes are combined with intravenous immunoglobulins, have been proposed to reduce anti-HLA antibody levels [1]. Although such strategies reduce anti-HLA antibody levels and facilitate transplantation in many cases [2,3,4,5,6], 25–30% of patients still retain many anti-HLA antibodies [7]. Within this context, alternative or additional strategies need to be developed.

Tocilizumab (TCZ) was the first humanized anti-interleukin-6 receptor subunit alpha monoclonal antibody licensed to treat rheumatoid arthritis and systemic juvenile idiopathic arthritis. TCZ can downregulate antibody production, orient naïve T-lymphocytes towards regulator T-lymphocytes, and reduce the number of Th_17_ lymphocytes [8]. A recent pilot trial that included 10 highly HLA-sensitized patients resistant to other desensitization strategies [5] suggested that the use of TCZ can reduce anti-HLA antibodies. However, only half of these patients were able to receive a transplant (5/10 patients) and one patient developed antibody-mediated rejection after transplantation [5]. In our recently published experience, TCZ as a desensitization therapy failed to induce a clinically significant reduction in anti-HLA antibody mean fluorescent intensity (MFI) [9], but limited B-cell maturation [10].

Such a variable response between patients may be related to the pharmacokinetic variability of TCZ. Indeed, the pharmacokinetics of TCZ [11], as that of numerous therapeutic monoclonal antibodies [12], is highly variable. For example, the range of TCZ concentrations measured at week 24 in patients suffering from rheumatological diseases was shown to be very large (from 0.2–35.4 mg·L^−1^) [13], with large coefficients of variation (CV) (132.6%) [11]. Moreover, several authors have reported a link between concentration and clinical efficacy in rheumatoid arthritis, even though such a relationship is still debated [11,14,15]. The pharmacokinetics of TCZ in dialyzed patients undergoing desensitization for kidney transplantation has not yet been explored but could be modified in end-stage renal disease due to, e.g., modifications of the distribution volume and/or non-specific proteic catabolism.

The primary objective of our study was to determine TCZ plasma trough concentrations and their inter- and intra-individual variabilities in kidney-transplant candidates treated for desensitization. In addition, the link between TCZ concentration and the evolution of anti-HLA antibodies was investigated. 

## 2. Materials and Methods

### 2.1. Study Design

Sensitized kidney-transplant candidates eligible for TCZ therapy for HLA desensitization were included in this pragmatic study. Plasma TCZ concentrations were determined from residual blood samples collected during routine care in 10 patients treated consecutively with TCZ between May 2017 and March 2019 in the Nephrology, Hemodialysis, Apheresis and Transplantation Department of Grenoble University Hospital. These samples were systematically handled just before each monthly TCZ infusion, in particular to check the absence of any inflammation (defined by C reactive protein level < 3 mg·L^−1^). Samples were stored at −80 °C in a biological sample collection (CRB04 AC-2017-2949). All patients provided written consent for the conservation and reuse of their samples. This retrospective monocentric study was approved by the Grenoble University Hospital review board (registration RnIPH 2020, protocol CINETOCI; CNIL number: 2205066 v 0).

Patients had received at least six TCZ infusions (8 mg/kg after a hemodialysis session over 30 min) every four weeks without the addition of any desensitization drug. The number of TCZ infusions could be extended beyond 6 months, as decided by the attending clinician. When TCZ cycles were completed, following our standard-of-care, patients received two infusions of rituximab and underwent apheresis for antibody removal until transplantation [9].

Demographic (age, gender, weight, height, and body-mass index), clinical (underlying disease, number of previous transplants), and biological (creatinine, Interleukin-6 (IL-6) and soluble IL-6 receptor (sIL-6R) levels) data were collected from our medical database.

### 2.2. Plasma TCZ Concentration Measurement

Plasma TCZ concentrations were determined by liquid chromatography-tandem mass spectrometry (LC-MS/MS), using a validated method previously described [11,16]. Briefly, following protein-G purification and trypsin digestion, two-dimensional liquid chromatography and mass-spectrometry were performed. Calibration curves ranged from 1.0 to 200.0 mg·L^−1^ and internal quality controls at 5.6, 45.0, and 150.0 mg·L^−1^ were used. The lower limit of quantification for TCZ was 1.0 mg·L^−1^. 

### 2.3. Plasma IL-6 and sIL-6R Assessment 

Plasma IL-6 and sIL-6R were quantified in plasma sampled immediately before the first TCZ infusion, at months 3 and 6 from the first TCZ infusion, using commercial ELISA kits (R&D system reference^®^). In some patients that benefited from more than 6 TCZ infusions due to persistence of DSA, additional IL-6 and sIL-6R measurements were performed when residual plasma samples were available.

### 2.4. HLA Antibody Analysis

HLA antibodies were evaluated for each TCZ sample using a Single Antigen test (Immucor^®^) on a Luminex^®^ platform, which enabled detection of immunoglobulin G (IgG) antibodies directed against class 1 or class 2 HLA antigens [9]. The results are expressed as MFI, with a positivity threshold for clinically significant antibodies at a MFI of 3000 [9].

We defined eliminated antibodies as HLA antibodies with an initial MFI > 3000 that was reduced to <3000 over the course of TCZ treatment. The number of eliminated antibodies was evaluated over the course of the full TCZ treatment and its link with the median TCZ concentration per patient was investigated. In addition, a fine-scale dose-response analysis was performed in which we used a Luminex^®^ evaluation of HLA antibody concentrations with each TCZ trough concentration measurement. We investigated the delta MFI between two samplings, n − 1 and n (for a given antibody, the difference between MFI at time n − 1 and MFI at time n), and correlated the resulting delta MFI with the TCZ trough concentration of sampling n.

### 2.5. Statistical Analyses

Continuous data are expressed as medians (25th–75th percentiles) and categorical variables as numbers (percentages). Inter and intra-individual CVs were calculated to assess inter- and intra-individual variability, respectively. Relationships between TCZ trough concentrations, TCZ trough concentrations adjusted on TCZ dose and demographic variables were investigated using a mixed-effects model in which the patient was a random factor. One model was generated per variable. We performed a log transformation of the TCZ concentration because the data were not normally distributed (Shapiro-Wilk test). An intra-patient correlation was used to study the link between TCZ concentration on one hand and IL6, sIL6-R levels on the other hand [17]. Finally, a mixed-effects model was also adjusted to predict MFI reductions depending on TCZ trough concentrations, using the patient as a random factor. In this multivariate mixed-effects model, the relative delta MFI was predicted based on initial MFI, HLA class and TCZ trough concentration. A *p*-value < 0.05 was considered statistically significant. Statistical tests were performed using Jamovi^®^ and the R statistical software.

## 3. Results

### 3.1. Baseline Characteristics of Patients

Ten patients awaiting a kidney transplant and for whom at least one HLA antibody had a MFI > 10,000 were treated by TCZ with available samples and were enrolled. The characteristics of this study population are presented in Table 1. The median number of TCZ infusions per patient was 9 (range: 7–10) and half of patients (5/10 patients) were able to access the kidney transplant.

### 3.2. Variability of Trough TCZ Concentrations

Seventy-four TCZ trough concentrations were determined among 10 patients. Overall, the TCZ trough concentration ranged from <1.0 to 52.5 mg·L^−1^, with a median of 25.6 mg·L^−1^ (25th–75th percentile: 13.2–35.3 mg·L^−1^). Two TCZ concentrations determined at M5 and M4 in two patients (P1 and P4) were very low (<1.0 and 1.1 mg·L^−1^), without an obvious cause having been identified. Considering all TCZ trough concentrations, the inter-individual CV was 55.0 % (51.9 % if the two abnormally low TCZ concentrations were excluded).

**Figure 1:**
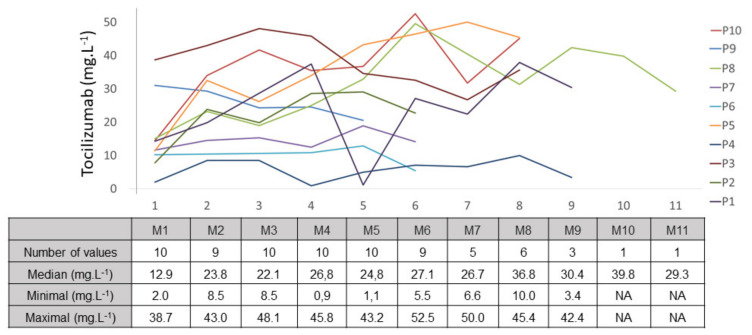
Temporal evolution of tocilizumab trough concentration. NA: not applicable, M: month after the first infusion of tocilizumab.

The median, minimal, and maximal TCZ trough concentrations, as well as the intra-individual CVs for each patient, are listed in Table S1. The intra-individual CV ranged from 16.0% to 54.0%, with a median of 33.0% (30.5% after exclusion of the two very low TCZ concentrations). The inter- and intra-individual variability of TCZ trough concentrations is illustrated in Figure 1.

In mixed-model analysis, no association between trough TCZ concentration and demographic characteristics (age, gender, weight, body-mass index) or the number of TCZ infusions was found, even when trough concentration was adjusted on TCZ dose.

### 3.3. Link between Tocilizumab Trough Concentrations, IL6 and sIL6-R Levels

**Figure 2:**
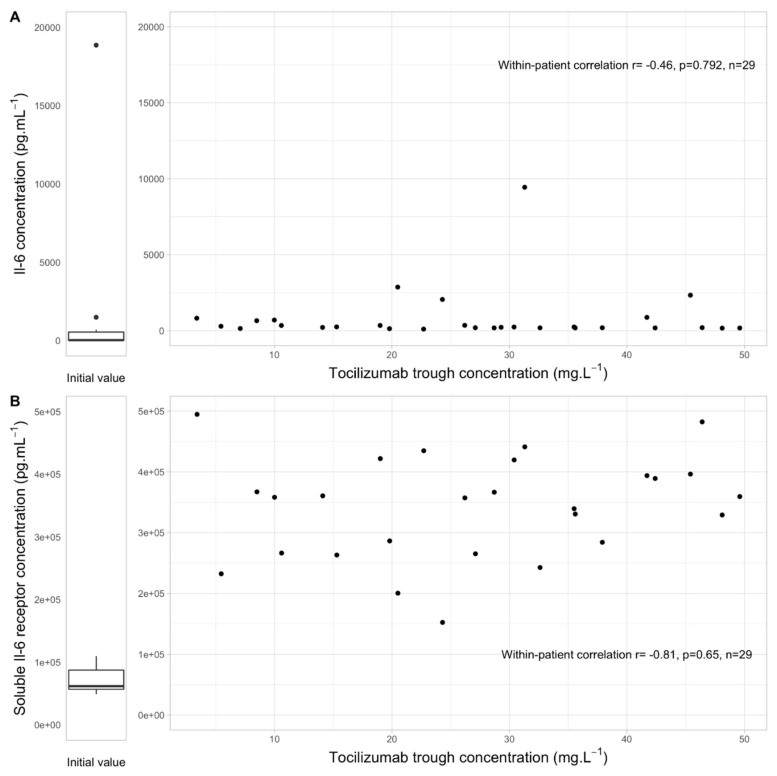
Within-patient correlation between tocilizumab trough concentration and IL-6 (**A**) or soluble IL-6 receptor (**B**) (*n* = 29), with initial IL-6 and soluble IL-6 receptor concentration before tocilizumab initiation.

No within-patient correlation between TCZ trough concentrations and IL-6 levels or sIL-6R was observed (Figure 2). 

### 3.4. Link between Tocilizumab Trough Concentrations and Anti-HLA Antibodies

The median TCZ concentration over the entire TCZ treatment period tended to be associated with the number of eliminated antibodies after the entire TCZ treatment (rho = 0.397, *p* = 0.083).

**Figure 3:**
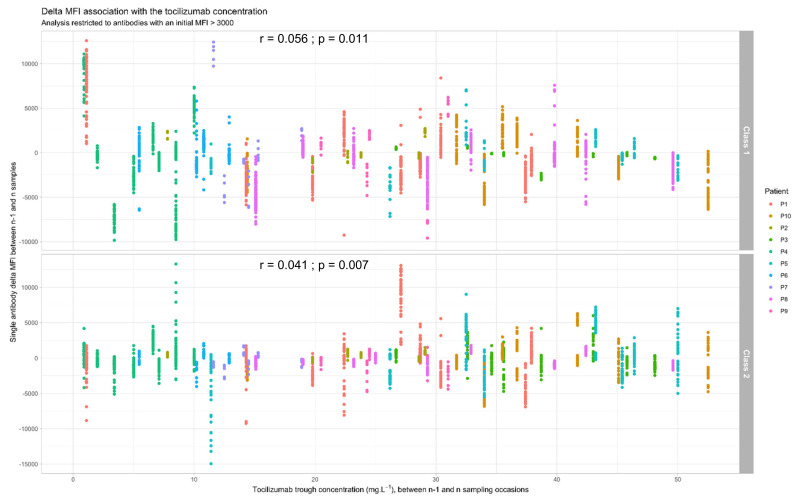
Concentration-effect relationship between tocilizumab trough concentration and the associated MFI reduction for individual antibodies. MFI: mean fluorescence intensity.

The delta MFIs between two successive samples were highly variable for a given TCZ trough concentration (Figure 3). They were very weakly correlated with the concomitant TCZ concentration, even if statistical significance was not reached for class 2 antibodies (Class 1: rho = 0.056, *p* = 0.011; Class 2: rho = 0.041, *p* = 0.07). In addition, TCZ trough concentrations were higher for eliminated class 2 antibody (reductions of MFI below the threshold of MFI = 3000) than for non-eliminated class 2 antibodies (TCZ trough concentration for eliminated antibodies: 28.6 [14.0–37.4] mg·L^−1^ versus 25.0 [12.9–35.5] mg·L^−1^ for non-eliminated antibodies; *p* = 0.001), whereas there was no difference for class 1 antibodies (*p* = ns).

However, relative delta MFIs between two successive TCZ administrations were not related (regression coefficient −0.004, *p* = 0.26) to TCZ trough concentration in a mixed-model (taking into account intra-patient variability) adjusting also for initial MFI (regression coefficient 3.28 × 10^−6^, *p* = 0.015), and HLA class (regression coefficient −0.016, *p* = 0.27).

## 4. Discussion

Our study describes, for the first time, TCZ plasma trough concentrations in patients awaiting kidney transplantation and treated for desensitization. 

TCZ trough concentrations measured in kidney-transplant candidates appeared to be higher than those reported for patients with rheumatoid arthritis treated with TCZ using the same regimen as in this study [11,13]. For example, Kneepkens et al. reported median TCZ concentrations of 3.4, 9.1, and 10.6 mg·L^−1^ in patients with rheumatoid arthritis at one, three, and six months after starting TCZ therapy, respectively [13], versus 12.9, 22.1, and 27.1 mg·L^−1^ recorded at the same timepoints in our study (Figure 1). These highly different exposure levels can be explained by a reduced target-mediated elimination in kidney-transplant candidates, associated with a less severe inflammatory status than that of patients with rheumatoid arthritis. Indeed, all patients of this study had C reactive protein level < 3 mg·L^−1^. However, such a hypothesis is not easily verifiable as, firstly, only trough concentrations were measured thus not allowing the determination of TCZ clearance, and secondly, various and not always comparable analytical methods (ELISA vs. LC-MS/MS) were used to quantify TCZ [14,18,19]. In addition, a modification of the TCZ pharmacokinetics by end-stage renal disease of patients included here (and their repeated dialysis sessions) could not be excluded, without any mechanism having been clearly identified.

The high inter- and intra-individual CVs of TCZ trough concentrations (i.e., 55.0% and 33.0%) that we found are in accordance with the variable TCZ concentrations determined for rheumatoid diseases [11,13,20,21]. However, the pharmacokinetic variability of TCZ in kidney-transplant candidates treated for desensitization appears to be less important than for patients with rheumatoid arthritis, as the inter-individual CV was lower than those previously described (55.0% in our study vs. 85% [14] and 132.6% [11] for patients with rheumatoid arthritis). Neither demographic parameters nor the number of TCZ infusions could explain the observed variability. However, the small number of measurements available in our study may have led to an underestimation of the pharmacokinetic variability of TCZ. Further research on larger cohorts is still needed. 

Concerning the association between TCZ concentrations and MFI, our data suggest a weak association between the number of eliminated antibodies (lowered to a MFI < 3000) and the median trough TCZ concentration. However, in the mixed-effect models accounting for intra-patient variability, no relationship between reduction of anti-HLA antibodies MFI and TCZ concentrations was found. These findings must be interpreted with caution given the low number of patients of our cohort. Since several studies have reported a concentration-effect relationship for TCZ in rheumatoid arthritis [13,15,20,22], further research conducted in larger cohorts are needed to explore such a concentration-effect relationship when TCZ is used for desensitization.

In addition, our results must be weighed up against the modest overall effect of TCZ as the single desensitization strategy [9,10]. Previous studies have suggested that the response to TCZ in rheumatoid arthritis is greater when sIL-6R levels are low [23]. Here, sIL-6R concentrations were relatively high, which may explain, at least in part, the marginal impact of TCZ on anti-HLA antibodies.

Our study has several limitations, including its retrospective design and the limited sample size. In addition, the pharmacokinetics of TCZ was only investigated by measuring TCZ trough concentrations, whereas additional pharmacokinetic parameters (e.g., area under the curve, clearance) should be investigated in future studies. However, this study is the first to explore exposure to TCZ when given as a desensitization strategy to the very specific population of highly sensitized kidney-transplant candidates.

## 5. Conclusions

This study demonstrates that TCZ trough concentrations in kidney-transplant candidates treated for desensitization are highly variable and are greater than in patients treated for a rheumatologic disease. Further research is needed to explore the concentration-effect relationship between TCZ exposure and MFI reduction.

## Figures and Tables

**Table 1:** Baseline characteristics of patients.

Characteristics at Tocilizumab Initiation	Values
Female	3 (30)
Age, years	52 (34–61)
Body-mass index, kg/m^2^	24.0 (22.2–26.5)
Weight, kg	70.5 (60.0–74.0)
Etiology of end-stage renal disease	
Glomerulonephritis	5 (50)
Uropathy	2 (20)
Others	3 (30)
Number of subjects with previous transplant	4 (40)
Number of tocilizumab infusions	9 (7–10)
Creatinine, µmol·L^−1^	867 (795–1058)
IL-6, pg·mL^−1^	26.8 (6.2–544)
sIL-6R, pg·mL^−1^	61,786 (56,820–87,334)
Number of antibodies with an initial MFI > 3000 per patient, class 1	25.5 (6.5–47)
Number of antibodies with an initial MFI > 3000 per patient, class 2	26.5 (12.2–37.8)

Data are presented as numbers (%) or medians (25th–75th percentiles). MFI: mean fluorescence intensity, IL: Interleukin, sIL-6R: soluble interleukin-6 receptor.

## Data Availability

Data are available upon reasonable request.

## Supplementary Materials

The following supporting information can be downloaded at: https://www.mdpi.com/article/10.3390/jcm11010091/s1, Table S1: Individual patient’s data for tocilizumab trough concentrations (TCZ Cmin) and donor-specific antibodies (DSA).
