# Supplementary material for: Tocilizumab Trough Levels Variability in Kidney-Transplant Candidates Undergoing Desensitization
Source: J Clin Med. 2021 Dec 24;11(1):91. doi: 10.3390/jcm11010091 (PMC8745611; doi:10.3390/jcm11010091)
Supplement: Supplementary file 1 [file jcm-11-00091-s001.zip › jcm-1474011-supplementary.pdf]

Table S1. Individual patient's data for tocilizumab trough concentrations (TCZ Cmin) and donor-specific antibodies (DSA).

Patient	Number of TCZ Cmin Measurement	Median TCZ Cmin $mg \cdot L^{-1}$	Min TCZ Cmin $mg \cdot L^{-1}$	Max TCZ Cmin $mg \cdot L^{-1}$	CV (%)	Number of DSA with a MFI > 3000 at M0	
						Class 1	Class 2
P1	9	27.1	1.1 *	37.9	48	48	41
P2	6	23.3	7.8	29.1	36	6	9
P3	8	37.2	26.7	48.1	19	5	30
P4	9	6.6	<1.0 *	10.0	54	35	42
P5	8	38.6	11.4	50.0	36	16	38
P6	5	10.6	5.5	12.9	27	48	16
P7	6	14.3	11.6	18.9	18	5	8
P8	10	30.3	15.1	49.6	35	73	23
P9	5	24.5	20.5	31.0	16	8	11
P10	8	36.1	14.4	52.5	31	44	37

CV: coefficient of variation, DAS: donor-specific antibodies, M: month, TCZ: tocilizumab. * indicates the two very low TCZ Cmin.
